# Retraction Notice to: Macrophage-Derived Slit3 Controls Cell Migration and Axon Pathfinding in the Peripheral Nerve Bridge

**DOI:** 10.1016/j.celrep.2023.112517

**Published:** 2023-05-05

**Authors:** Xin-peng Dun, Lauren Carr, Patricia K. Woodley, Riordan W. Barry, Louisa K. Drake, Thomas Mindos, Sheridan L. Roberts, Alison C. Lloyd, David B. Parkinson

(Cell Reports *26*, 1458–1472.e1–e4; February 5, 2019)

This article has been retracted at the request of the authors. This paper reported a role for Slit-Robo signaling in the control of axon guidance during peripheral nerve regeneration. The first author has admitted, orally and in writing, to the fabrication of data, unknown to other authors, and is solely responsible for this fabrication. Based on reports from the scientific community, the authors began an investigation of data and procedures in the paper. They concluded that the paper contains fabrications of data in Figure 2, panels K and O. For panel K, the image for Robo2 was copied from another publication by the first author (Figure 12 of Carr et al., 2017, *PLoS ONE 12*, e0172736, https://doi.org/10.1371/journal.pone.0172736). For panel O, data were taken from other PCR experiments. While the authors have reproduced and confirmed the data for panel O, given the circumstances, they believe that the most responsible course of action is to retract the paper. The authors sincerely apologize to the scientific community for any inconvenience resulting from the publication and retraction of this manuscript.

